# Enhancing radiative efficiency in MHD micropumps using plasma-infused hybrid bioconvective nanofluids for advanced radiative oncology at tertiary level

**DOI:** 10.1038/s41598-023-45513-5

**Published:** 2023-10-27

**Authors:** Abyaz Abid, A. K. Azad, Arafat A. Bhuiyan

**Affiliations:** Mechanical and Production Engineering, Islamic University of Technology (IUT), Board Bazar, Gazipur, 1704 Bangladesh

**Keywords:** Nanobiotechnology, Mechanical engineering, Oncology, Energy science and technology, Engineering

## Abstract

This research paper investigates the optimization of radiation performance of a plasma-based bioconvective nanofluid integrated Magneto-hydrodynamic (MHD) micropump for radiative oncology. It addresses a literature gap by analysing the radiative impact of blood-based hybrid nanofluids in MHD micropumps. Three blood-based bio-convective radiating hybrid nanofluids—blood—Pt, blood—Au and blood—MWCNT are studied to understand their radiation behaviour in MHD pump while being employed as transportation medium. The investigation employs two non-dimensional parameters, namely Rd (Radiation number) and Ha (Hartmann number), to examine the fluid dynamics, magnetic characteristics, and electrical properties of the MHD micropump. The temperature gradient, velocity distribution, and pressure drop along the flow channel are examined within the specified range of Rd and Ha. Magnetic flux density (MFD) and electric flux intensity (EFI) are evaluated to understand nanoparticle behaviour during drug delivery and blood transportation. Findings highlight that MWCNT and Pt are the most efficient bioconvective nanoparticles for plasma transportation under high radiative conditions. MWCNT-based blood flow exhibits desirable characteristics, including sufficient intake pressure of 4.5 kPa and minimal relative pressure drop of 34%. Coherence between radiation flux and electromagnetic flux reduces pumping power and ensures uniform heat dissipation for improved drug delivery. Au nanoparticles provide moderate magnetic flux density with least fluctuation within the range of Ha and Rd number (2.57 T to 4.39 T), even in highly radiative environments (such as—Rd = 4, Rd = 5), making them suitable for applications like embedded chemotherapy or cell treatment. Au nanoparticles maintain moderate electrical flux intensity with a minimal drop of 16nA, particularly at higher radiative environments influenced by the Radiation number (Rd = 4 to Rd = 5) while Ha values from Ha = 2 to Ha = 4. Conclusively, it has been identified that MWCNT and Au are superior nanofluids for advanced radiative oncological treatments. These nanofluids have the potential to enhance plasma transportation, thermal regulation, and aetilogical disease management. The present study provides significant findings on enhancing the radiation performance in MHD micropumps through utilization of blood-based hybrid nanofluids, thereby offering potential advantages to the domain of biomedical engineering.

## Introduction

When a magnetic field and an electrically conducting fluid interact, a phenomenon known as magnetohydrodynamics (MHD) takes place. The fundamental tenet of MHD is that a magnetic field can cause an electrical current to flow in a conducting fluid, creating a force that can be used to move the fluid or produce electricity. MHD is used in a variety of applications, including the creation of electricity in power plants and the design of plasma thrusters for spacecraft^1–3^. The potential of MHD to increase energy harvesting and ease pressure on conventional fuels is one of its most exciting implications. The amount of energy that can be extracted from a given amount of fuel is greater when using MHD generators to convert heat energy into electricity than it is with conventional steam turbines^4^. This is so that they can get more energy out of the same amount of fuel because MHD generators can work at higher temperatures and pressures. MHD generators can generate electricity more effectively than other types of generators, which lowers the overall amount of fuel required to produce a given amount of power^5,6^. MHD is also used in the creation of spacecraft propulsion systems. A lightweight, highly effective propulsion system that can function in the vacuum of space can be built using MHD thrusters. This is because MHD thrusters rely on the interaction between a magnetic field and plasma to produce thrust rather than any external propellant. They are therefore perfect for lengthy space missions where weight and fuel efficiency are important considerations^7–9^.

Micropumps are micro level pumps whose working are based on Magnetohydrodynamic (MHD) principles and are utilized to transport fluid. Magnetohydrodynamic (MHD) micropumps function based on the principle of Lorentz force generation through the application of a magnetic field, which results in the directed propulsion of fluid. The Lorentz force arises due to the interaction between a magnetic field and a conductive fluid carrying an electric current. The cross-product of the magnetic field and the current density results in a Lorentz force that induces a motion of the fluid in a direction that is orthogonal to both the magnetic field and the current density^10–12^.

The popularity of MHD micropumps has increased in recent times owing to their capacity to pump small volumes of fluid with high precision, without necessitating the use of moving parts. MHD (magnetohydrodynamic) micropumps exhibit low acoustic emissions, high energy efficiency, and necessitate minimal upkeep^13–15^. Micromagnetic hydrodynamic (MHD) pumps exhibit a diverse array of potential applications, such as microfluidic lab-on-a-chip platforms, drug administration apparatuses, and electronic component cooling mechanisms. These pumps find utility in biomedical settings for the purposes of drug administration, blood examination, and ailment identification^16,17^. Magnetohydrodynamic (MHD) micro pumps have been employed for targeted drug delivery within the human body, thereby mitigating undesired side effects and enhancing the therapeutic efficacy of treatments. As an instance, they can be employed for the administration of insulin to individuals with diabetes or chemotherapeutic agents to individuals with cancer^18–20^. Moreover, Magnetohydrodynamic (MHD) pumps possess the capability of amalgamating with microfluidic contraptions to fabricate lab-on-a-chip arrangements, that enable the handling and examination of diminutive fluid quantities^21,22^. In general, MHD micro pumps have demonstrated significant efficacy and potential for diverse biomedical applications, thereby facilitating the advancement of more sophisticated microfluidic apparatus.

Hybrid bioconvective nanofluids that are blood-based have gained prominence in the field of research owing to their distinct characteristics and possible medical uses. Blood-based nanofluids are preferred due to their biocompatibility and hemocompatibility, which enable them to circulate within the bloodstream without inducing any deleterious effects. Nanofluids composed of blood are utilized in diverse scientific applications such as drug transportation, biosensing, and imaging^23–25^. The unique feature of blood-based nanofluids in the medical domain is their capacity to selectively target particular tissues or cells within the human body. One such implication of blood-based hybrid nanofluids is presented in Refs.^26,27^ where Ag-Au augmented nanoparticles are used in the blood base fluid through cosine stenosis in the presence of angular magnetic field. Such research resulted in improved heat transfer within the blood due to the use of crossing nanoparticles. Gold and silver nanofluids can be modified with specific biomolecules such as antibodies and antimicrobial peptides respectively, to selectively target cancer cells and combat infections^28,29^. Mubashir Quayyum et al. examined behaviour of a combined two different nanoparticles i.e. UO_2_ (Uranium dioxide) and MWCNT (multi walled carbon nanotube) with blood as the base fluid for two rotating stretching discs with convective boundary^30,31^. Results showed that skin friction and heat transfer rate is profoundly high at the convective boundary layer and low at the non-convective layer. However, mass transfer exhibited opposite traits to that of heat transfer rate. Carbon nanotube nanofluids derived from blood have been utilized in the fields of biosensing and imaging^32,33^. Silver nanofluids derived from blood have been utilized for the purpose of promoting wound healing and exhibiting antimicrobial properties^34–36^. Furthermore, these nanofluids have exhibited encouraging outcomes in the management of neurological conditions such as Alzheimer's disease and Parkinson's disease. Blood-based nanofluids containing gold, carbon nanotubes, and silver have demonstrated promising applications in drug delivery, biosensing, wound healing, and antimicrobial therapy.

Radioactive analysis of such bio generative hybrid nanofluid is crucial because ionizing radiation can harm biological systems, such as blood, and it's crucial to comprehend how these nanofluids might be impacted by radiation exposure. Also, understanding the radiation analysis of blood-based hybrid bioconvective nanofluids can lead to better understanding of how these nanofluids might be used in radiation therapy for cancer and other medical applications^37–39^. Gold nanoparticles associated with DNA, RNA protein when used to treat cancer cells and fatal diseases are prone to radiation effect at the slip boundary conditions. Outcomes of the study are provided both in graphical and analytical form^40,41^. In order to study the radiation efficiency of incorporated Ag and Au nanoparticles in blood for drug delivery Hasan et al. conducted numerical investigation to discover how the coated boundary layer regime is shaped up due to the radiation effect while the results are presented in terms of temperature and velocity profile^42,43^. Steady, laminar and magnetohydrodynamic flow of Casson nanoparticle (blood-based Au and Ag nanofluid) coupled with Hall current, Joule heating under modified Darcy Law—is extensively studied in Refs.^44–46^ and propagated outcomes in the domains of tumour therapy, biomedical imaging and cancer therapy. Illustration of momentum and energy profiles are done with respect to various physical constraints in order to inquire about the radioactive impact in Refs.^47–49^ which particularly focuses on the flow of blood-based CNT nanofluid particles with variable viscosity^50,51^.

Although a lot of scientific contribution has been provided to radiative characteristics of different nanofluids, MHD flow through various medium, feasibility of micropump for biomedical applications and so on; however, a comprehensive demonstration of radioactive impact analysis of such blood-based hybrid nanofluids when used within the microchannel of MHD micropump—still lacks in available literature. Due to incessant usage as an impactful biomedical micro equipment, its propensity in the field of cancer treatment while different blood based bioconvective nanofluids are used as transporting medium remains an ideal field to explore further. And therefore, the proposed paper seeks to explore this research gap and signifies the outcome as contribution to the field of biomedical engineering for transporting blood and delivering drug in human body. In order to inspecting the radiation behaviour of MHD pump three different blood-based bio-convective radiating hybrid nanofluids are used as transport medium—*BL* + *Pt (blood-based platinum nanofluid), BL* + *Au (blood-based gold nanofluid)* and *BL* + *MWCNT (blood-based carbon nanotube nanofluid).* Due to their self-radiating nature, the blood based nanofluids are coupled with an external heat source (*Cu*) and heat sink (*Al*) to protein and amplify the outcome of the computational study. Two dimensionless parameters Rd (*Radiation number*) and Ha (*Hartmann number*) are introduced in this paper in order to present the outcomes under the scope of three fundamental criteria—fluid properties, magnetic properties and electrical properties of the MHD micropump. To observe the deviation of fluid properties such as—temperature gradient, velocity distribution and pressure drop along the flow channel of the MHD pump, the dimensionless numbers are considered within the range from one to five and one to four for Rd and Ha number respectively. The other two rudimentary properties—magnetic flux density (MFD) and electric flux intensity (EFI) are also being shown within the given range to understand how the nanoparticles behave inside human body during drug delivery and blood transportation.

## Materials and methods

### Domain setup

**Figure 1:**
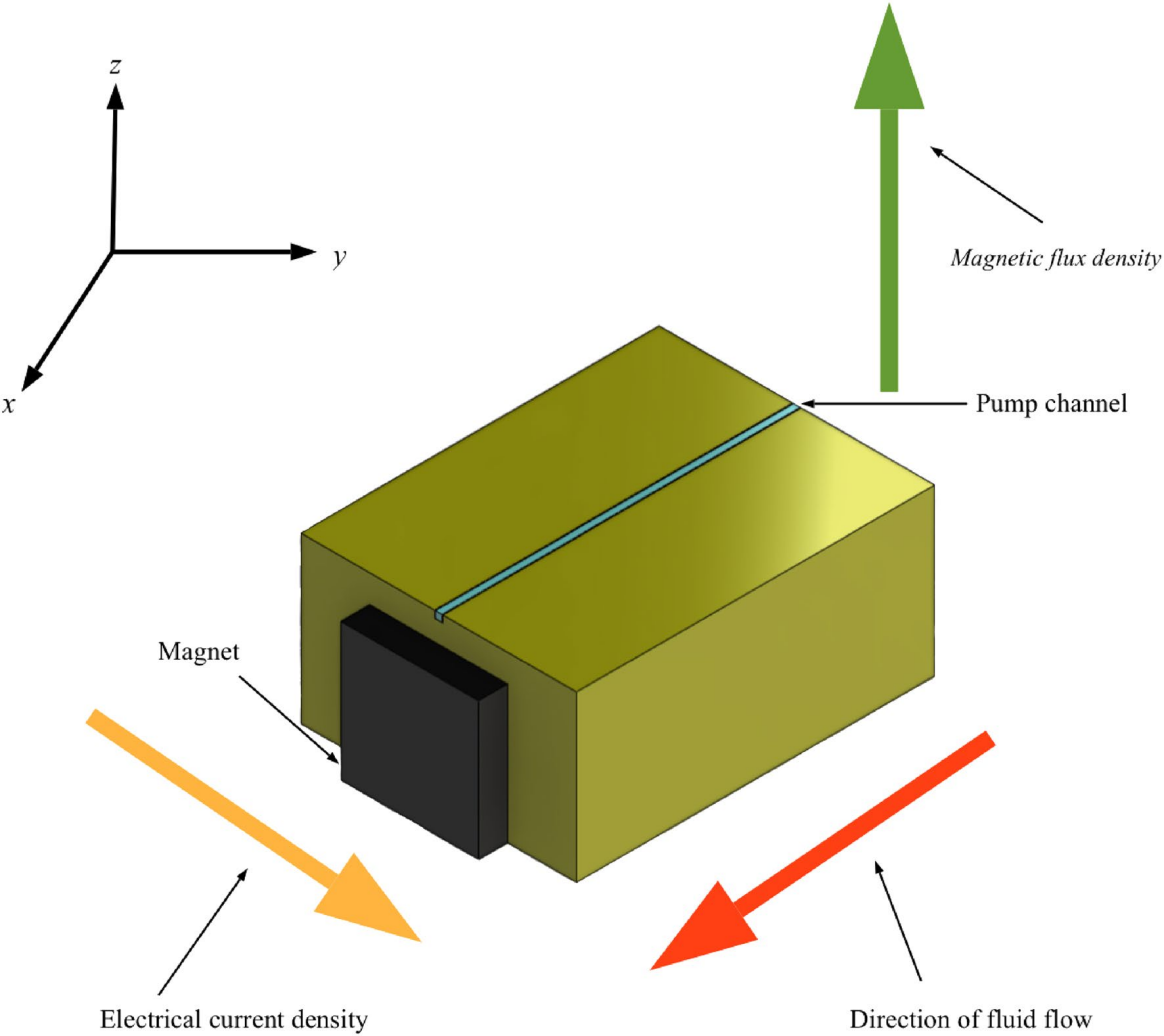
A design concept for the proposed MHD micropump.

**Figure 2:**
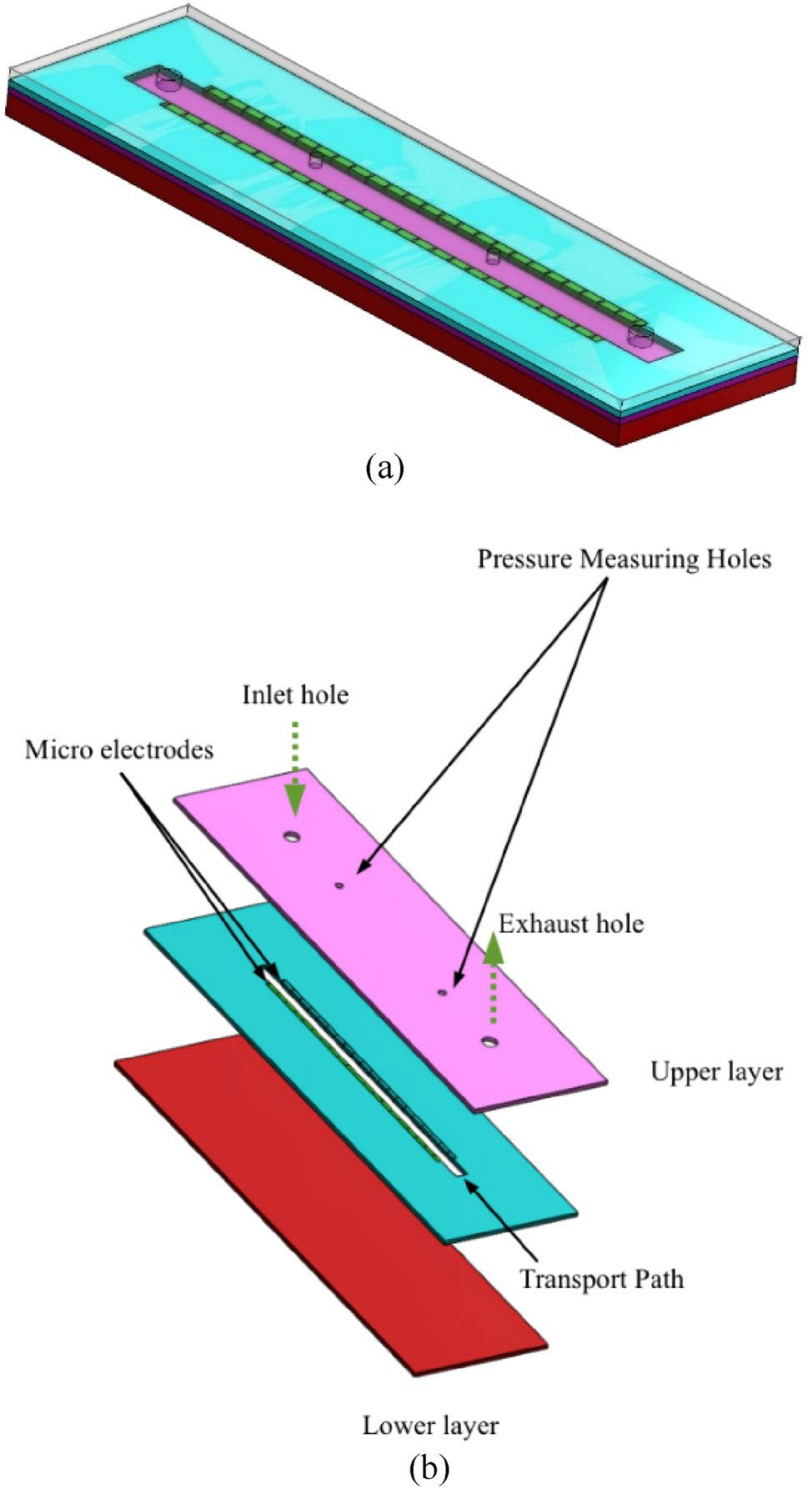
An isometric view of the distinct components of the MHD micropump: (**a**) A complete setup, and (**b**) an exploded view of the complete setup.

**Figure 3:**
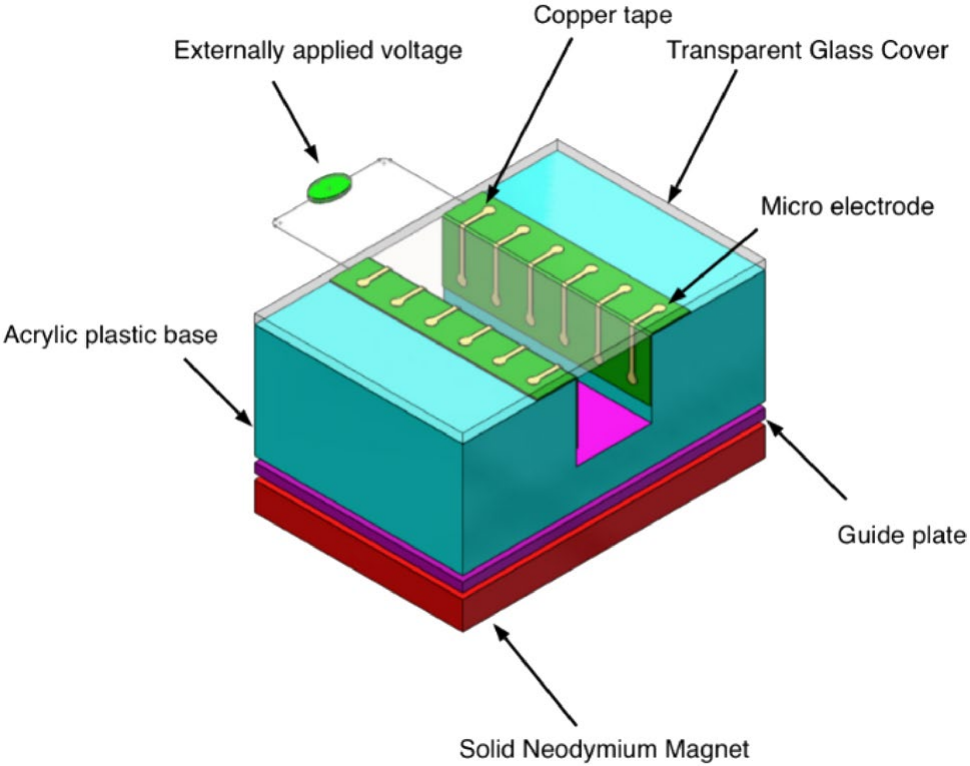
Cross sectional view of the MHD Micropump.

A conceptual schematic of Magneto Hydrodynamic (MHD) Micropump has been provided in Fig. 1. The pump which is simply a combination of magnetic field, electric field and fluid transporting channel works based on the generated Lorentz force. The illustration provides directional guidance for the working MHD pump which has fluid flow, magnetic flux density and electric field intensity along *x*, *z* and *y*-direction respectively. The developed Lorentz force due to orthogonally applied electrical and magnetic force thrusts the fluid through the pump channel without any mechanical intervene. Figure 2a provides a complete set up for an experimental MHD micropump and Fig. 2b displays an exploded view of the same pump. The pump is mainly consisting of three distinct layers—the lower layer holds magnetic source (in this case the Neodymium solid magnet) while the middle layer holds the fluid transport path of the pump. The upper most layer is the layer which protects the pump channel with a transparent glass cover and facilitates pressure measurement by holding two pressure measuring holes. There is also one inlet port for allowing the fluid into the pump and an outlet port to discharge the volume flow. Finally, Fig. 3 captures a cross sectional view of the same MHD micropump to demonstrate the parts at micro level—a guide plate to separate out the magnet source (at lower layer) and acrylic plastic base (at middle layer), a thin copper film as grounding for setting series of copper micro electrode and finally an external voltage to provide just enough amount of current for the pump to work. A transparent glass cover is to protect the core flow channel of the pump from any external harm without disallowing outer supervision.

### Underlying presumptions

Most of the basic principles of MHD phenomena are implied by important physical parameters in the computational model. Yet the most accurate form of the numerical domain is underpinned by some necessary assumptions. The assumptions that support the ongoing investigations are presented below—

- The MHD micropump is devoid of ideal MHD effect due to geometrical complexity.
- Dissipative heat transfer terms are included in the methodology section of the paper as there is generation of entropy due to Ohm’s Law.
- As the structural dimensions of MHD micropump is much comparable to that fluid particlez, continuum theory is not applied for this domain.
- Micropolar fluid theory is considered for the study.
- The transporting fluid flow is assumed to be two dimensional, laminar, incompressible and unidirectional.
- Power-law model is sincerely negotiated for the study as the fluid of interest is already determined.

### Governing equations

The fundamental framework of the forums for applied mathematical modelling can be categorized into three distinct groups: 1. Electromagnetic systems, 2. Conventional fluid dynamics systems, and 3. Thermal systems.

#### Electromagnetic equations

The electromagnetic characteristics of the MHD micropump domain are determined by three fundamental parameters: current density, magnetic flux density and electric field. Despite the electrical conductance permitted through the micropump channel, the resulting magnetic effect on the overall geometry is disregarded due to the negligible value of the associated magnetic Reynolds number (*Re*), as widely reported in existing literature^52^.

Maxwell equations:

1 $$\nabla .D=\rho$$

2 $$\nabla . B=0$$

3 $$\nabla \times E= -\frac{\partial B}{\partial t}$$

4 $$\nabla \times H=J+\frac{\partial D}{\partial t}$$

Ohm’s Law:

5 $$J= \delta \left(E+u\times B\right)= \delta (-\nabla \varnothing +u\times B)$$

By combining with Ohm’s Law:

6 $$J=\delta (E+v\times B)$$

The Lorentz force:

7 $${F}_{L}=(J\times B)$$

#### Conventional fluid dynamic equations

Fluidic part of the modelling system is consisting of simple continuity equation, momentum equation and energy equations^53^.

Continuity equation:

8 $$\frac{\partial u}{\partial x}+\frac{\partial v}{\partial y}=0$$

Momentum equations:

9 $$\frac{\partial u}{\partial t}+u\frac{\partial u}{\partial x}+v\frac{\partial u}{\partial y}= -\frac{1}{{\rho }_{nf}}\frac{\partial p}{\partial x}+\frac{{\mu }_{nf}}{{\rho }_{nf}}\left(\frac{{\partial }^{2}x}{\partial {x}^{2}}+\frac{{\partial }^{2}u}{\partial {y}^{2}}\right)$$

10 $$\frac{\partial v}{\partial t}+u\frac{\partial v}{\partial x}+v\frac{\partial v}{\partial y}= -\frac{1}{{p}_{nf}}\frac{\partial p}{\partial x}+\frac{{\mu }_{nf}}{{\rho }_{nf}}\left(\frac{{\partial }^{2}v}{\partial {x}^{2}}+\frac{{\partial }^{2}v}{\partial {y}^{2}}\right)+ \frac{{\sigma }_{nf}{B}_{0}^{2}}{{\rho }_{nf}}v+\frac{{\left(\rho \beta \right)}_{nf}}{{\rho }_{nf}}g({T-T}_{c})$$

Energy equations:

11 $$\frac{\partial T}{\partial t}+u\frac{\partial T}{\partial x}+v\frac{\partial T}{\partial y}= {\alpha }_{nf}\left(\frac{{\partial }^{2}T}{\partial {x}^{2}}+\frac{{\partial }^{2}T}{\partial {y}^{2}}\right)-{\frac{1}{(\rho {c}_{p})}}_{nf}\frac{\partial {q}_{R}}{\partial y}$$

#### Thermal system equations

The thermal energy equation^54^ is presented is below here:

12 $$\rho c\left(\frac{dT}{dt}+\mu \nabla T\right)=K{\nabla }^{2}T+ \frac{|{J|}^{2}}{\delta }$$

The thermal system of the domain is interconnected with the flow of electric current through fluid medium integrated in the MHD micropump.

### Boundary conditions

Analogous to the governing equations of the modelling system, boundary conditions are imposed to incorporate the fundamental fluid flow throughout the domain, as well as the interplay of electricity, magnetism, and thermal system with the wall of the pump. The prescribed conditions that define the interface between the fluid and the pump are presented as follows.

#### Boundary conditions for fluid-pump interaction

Boundary conditions at inlet^55^:

13 $$\frac{{\partial }_{{u}_{x,in}}}{{\partial }_{x}}= {u}_{z,in}= {u}_{y,in}=0$$

14 $${P}_{in}=0$$

15 $${T}_{in }= {T}_{0}$$

Boundary conditions at pump wall^56^:

16 $${u}_{wall}=0$$

17 $$\frac{{\partial }_{{P}_{wall}}}{{\partial }_{n}}=0$$

18 $${T}_{wall}= {T}_{0}$$

19 $${\frac{\partial \varphi }{\partial n}}_{wall}=0$$

Boundary conditions at pump outlet^57^:

20 $$\frac{{\partial }_{{u}_{out}}}{{\partial }_{t}}+ {u}_{x,out}\frac{{\partial }_{{u}_{out}}}{{\partial }_{x}}=0$$

21 $${P}_{out}=0$$

22 $$\frac{{\partial }_{{T}_{out}}}{{\partial }_{x}}=0$$

23 $${\frac{\partial \varphi }{\partial n}}_{out}= 0$$

Boundary conditions at electrode surface^57^

24 $${\varphi }_{anode}= {V}_{input}$$

25 $${\varphi }_{cathode}=0$$

#### Boundary conditions for basic fluid flow through MHD pump

For *t* = *0: u* = *v* = *0, T* = *0, p* = *0.*

For *t* > *0: u* = *v* = *0, T* = $${T}_{h}$$ at* y* = *0,* $$0.25L\le x\le 0.65L$$

$$u= {u}_{0}, v=O, T={T}_{c}$$ at *y* = *L; 0* $$\le x \le L$$

*u* = *v* = *0,* $$\frac{\partial T}{\partial N}=O$$ at *x* = *0, L; 0* $$\le y \le L$$

*u* = *v* = *0,* $$\frac{\partial T}{\partial N}=O$$ at *y* = *0, 0* $$\le x \le 0.25L, 0.65L\le x \le L$$

#### Boundary conditions for converging–diverging channel

An intriguing facet of the computational analysis involves examining the effects of channel shape contraction and expansion on the flow of blood-based hybrid nanofluids through various arteries and veins within the human body. The realization of this effect via the MHD pump involves the utilization of Jeffery-Hamel dimensional analysis, which closely approximates numerical output. This method is a direct application of the Tiwari-Das nanofluid technique^56^.

26 $$\left\{\begin{array}{c}\frac{\partial u}{\partial \theta }=0, \frac{\partial T}{\partial \theta }=0, u=U\\ u= -{N}_{1}{v}_{nf}\frac{\partial u}{\partial \theta }, T= \frac{{T}_{w}}{{r}^{2}}- {D}_{1}\frac{\partial T}{\partial \theta }\end{array} |\begin{array}{c} at \theta =0\\ at \theta = \alpha \end{array}\right\}$$

### Dimensional analysis

The variables were transformed into non-dimensional form to decrease the quantity of available variables and to simplify the governing equations into a non-dimensional form. Non-dimensionalization has been performed for the variables of both the fundamental fluid flow and flow through the converging–diverging channel, as presented below^58^.

27 $$Y= \frac{y}{L}, X= \frac{x}{L}, U= \frac{u}{{u}_{0}}, V= \frac{v}{{u}_{0}}, P= \frac{p}{{\rho }_{nf}{u}_{0}^{2}}, \theta = \frac{T- {T}_{C}}{{T}_{h}- {T}_{C}}$$

28 $$\eta = \frac{\theta }{\alpha }, f\left(\theta \right)=ru\,\left(r,\theta \right), \Theta \left(\eta \right)= {r}^{2}\frac{T}{{T}_{w}}$$

### Nanofluid properties

As previously stated, four distinct hybrid nanofluids composed of blood have been utilized as the fluid medium for the micropump. Formulas are employed to incorporate various fluidic properties, including specific heat (*C*_*p*_), viscosity (*μ*_*static*_), effective density (*ρ*_*nf*_), thermal expansion coefficient (*β*_*nf*_), and thermal diffusivity (*α*_*nf*_), of nanofluids into the modelling system^59^.

29 $${\rho }_{nf}=\left(1-\varphi \right){\rho }_{nf}+ \varphi {\rho }_{s}$$

30 $${(\rho {C}_{p})}_{nf}= \left(1-\varphi \right) {(\rho {C}_{p})}_{f}+ \varphi {(\rho {C}_{p})}_{s}$$

31 $${(\rho \beta )}_{nf}=\left(1-\varphi \right){\rho \beta }_{f}+ \varphi {(\rho \beta )}_{s}$$

32 $${\mu }_{static}= \frac{{\mu }_{f}}{{(1-\varphi )}^{2.5}}$$

33 $${\alpha }_{nf}= \frac{{k}_{nf}}{{(\rho {C}_{p})}_{nf}}$$

The current investigation incorporates both the static and Brownian thermal conductivities of the transport fluid particles to examine the heat transfer efficiency of the nanofluids within the micropump. The model input includes *k*_*eff*_ for the ultimate goal.

34 $${k}_{static}= \frac{{k}_{s}+2{k}_{f}-2\varphi ({k}_{f}-{k}_{s})}{{k}_{s}+ 2{k}_{f}\varphi ({k}_{f}-{k}_{s})}{k}_{f}$$

35 $${k}_{Brownian}= \frac{\varphi {\rho }_{p}{C}_{p,p}}{2}\sqrt{\frac{2{K}_{B}{T}_{ref}}{3\pi d{\mu }_{static}}}$$

36 $${k}_{eff}={k}_{static}+ {k}_{Brownian}$$

Similar to the study of thermal conductivity, the effective viscosity of nanofluids is also being studied.

37 $${\mu }_{eff}= \frac{{\mu }_{f}}{{(1-\varphi )}^{2.5}}+ \frac{{k}_{Brownian}}{{k}_{f}}\times \frac{{\mu }_{f}}{{Pr}_{f}}$$

Thermo-physical properties of the bioconvective hybrid nanofluids are presented in Table 1 for conducting the computational workload. Table 2 showcases the values of volume fractions taken for all the blood-based hybrid nanofluids.

**Table 1:** Thermo-physical characteristics of the selected bioconvective hybrid nanofluids for conducting computational workload^53,60,61^.

Thermo-physical characteristics	Base fluid (Blood)	Pt nanoparticels	Au nanoparticles	MWCNT nanoparticles
*C*_*p*_ (J/kg. K)	3617	133	129.1	796
$\rho$ (kg. m^3^)	1050	2145	19,300	1600
$\sigma (S/m)$	1.33	9.5 $\times 10$^6^	4.5 $\times 10$^7^	5 $\times 10$^7^
$\beta$ (1/K)	0.18	15.3 $\times 10$^−5^	1.42 $\times 10$^−5^	44 $\times 10$^−5^
*k* (W/m. K)	0.52	210	320	3000

**Table 2:** Volume fractions of the proposed three blood based hybrid nanofluids^62^.

Pt nanoparticles	0.035
Au nanoparticles	0.02
MWCNT nanoparticles	0.03

### Mesh generation and convergency test

**Figure 4:**
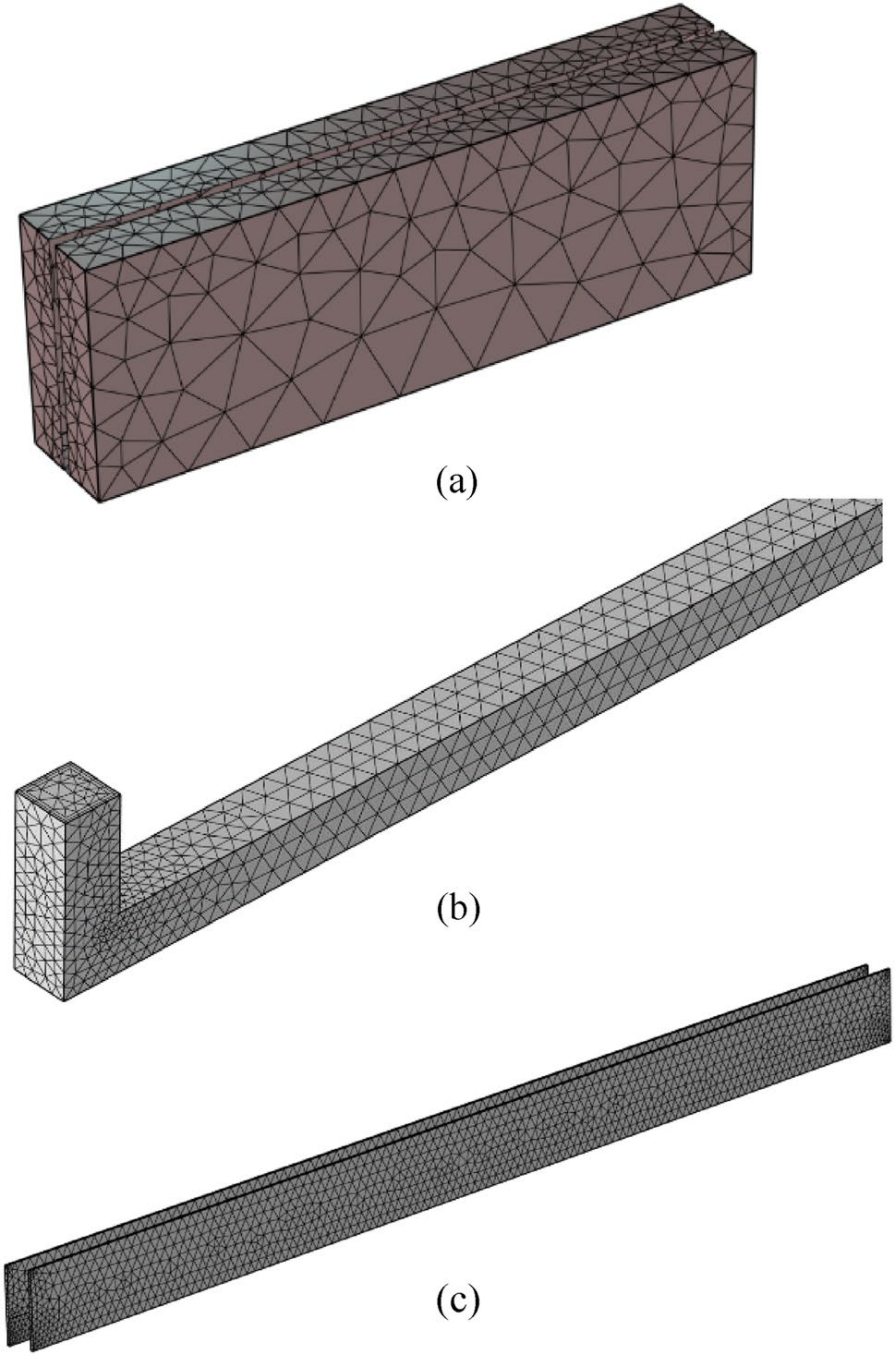
Different viewpoints of distinct mesh components of the proposed MHD pump: (**a**) Neodymium magnet, (**b**) Fluid domain, and (**c**) Cu electrodes.

The complete magneto-hydrodynamic (MHD) micro pump system has been partitioned into four distinct subsystems, namely: 1. Static Magnetic domain, 2. Electrical current domain, 3. Plastic domain (as pump channel base), and 4. Fluid system domain. To enhance the computational efficiency of the MHD system, all subsystems, except for the acrylic plastic domain, have been discretized into free tetrahedral and triangular elements. Figure 4 represents different views of the MHD micro pump with all of its distinct meshed parts. Close observation opens up the case that—coarse mesh is applied for the magnetic portion of the pump whereas fine mesh is implemented for the thin electrical Cu wire. A finer mesh has applied for the fluid region to study the heat transfer, velocity, pressure and temperature insight of the medium.

**Figure 5:**
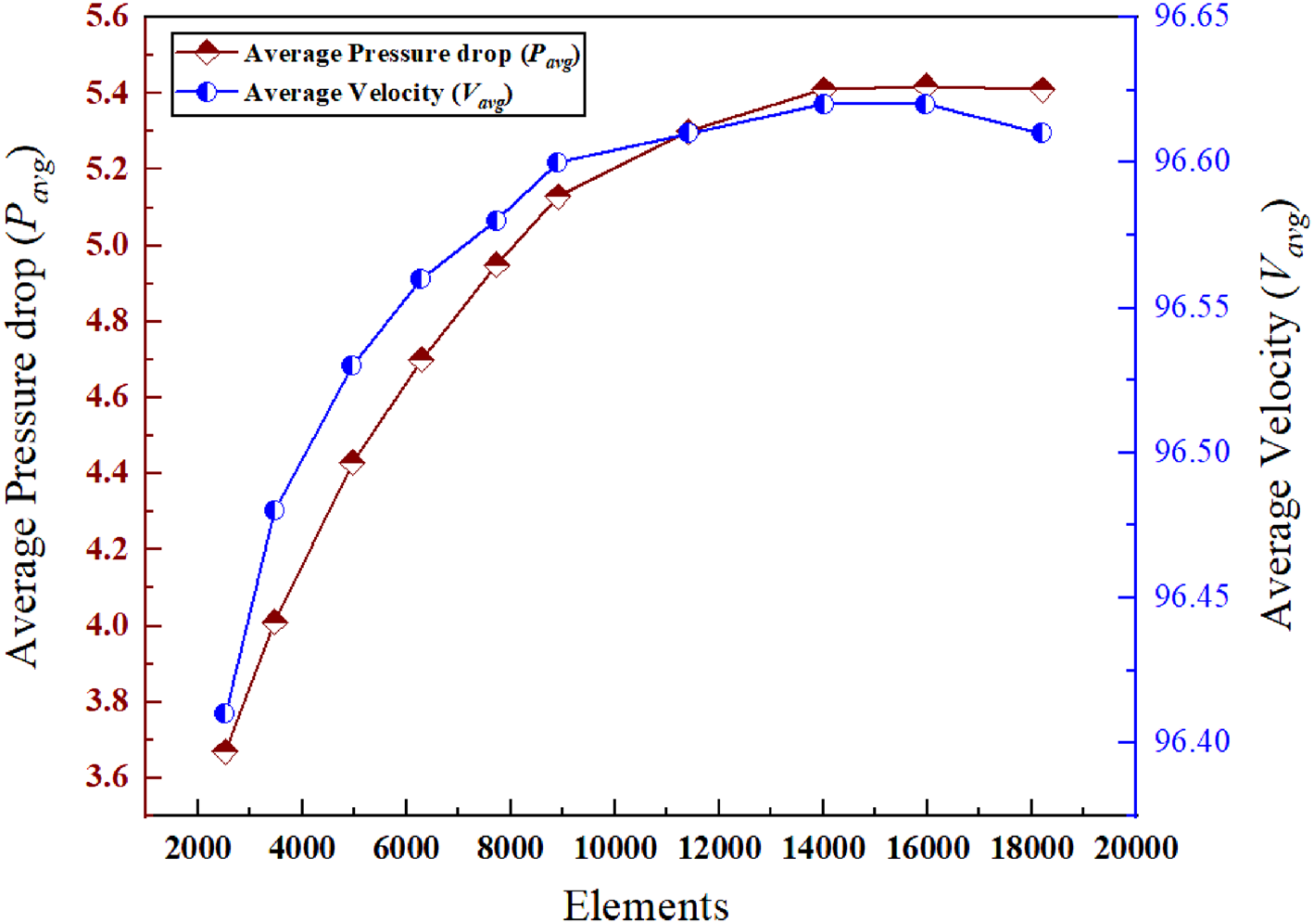
Grid convergency test for the represented value at *Ri* = 3, *Ha* = 2, *Rd* = 2 and $\varphi$ = 0.04.

In order to determine when the applied numerical process reached convergency, a grid independence test is performed. The convergency test is primarily conducted to lessen the computational load and processing time of commercially available software. Figure 5 represents guided convergence test of the paper for constant values of Ri = 3, Ha = 2, Rd = 2 and $\varphi$ = 0.04 while two rudimentary parameters—average pressure drop (*P*_avg_) and average flow velocity (*V*_*avg*_) along the pump channel are chosen to illustrate insights of the test. It is to be found from the paper that the ultimate grid independence of the numerical procedure is achieved for element number around 21,000. Trend lines of both the parameters show steady increase with the element number while the average magnetic flux intensity exhibits a more protean pattern compared to the magnitudes of average electrical current intensity.

### Validation

**Figure 6:**
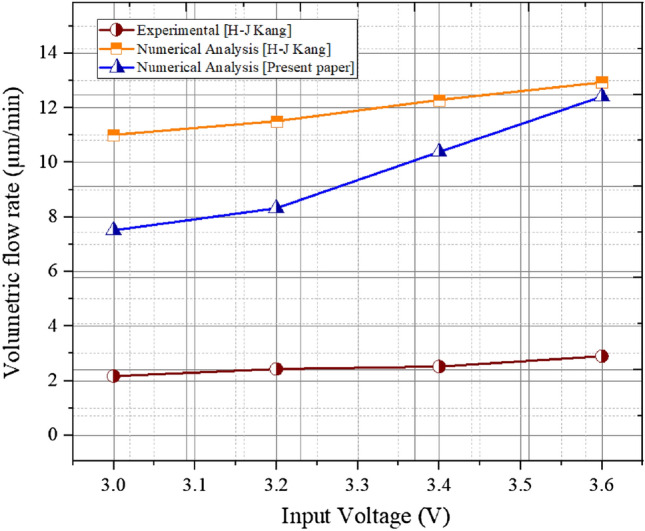
Comprison among the experimented and numerical work done in H-J Kang [3] with numerical work done in this paper.

Authenticity of the proposed numerical modelling system has been investigated against the experimental and numerical outcomes of Ho-Jin Kang et al.^52^. Figure 6 provides illustration of the trending lines for volume flow rate of transport medium against applied input voltage. For understanding the flow rate of the liquid, magnitude of the applied voltage has been steadily increased but within a limited range. The graph exhibits that numerical outcomes are on average around 8% deviated from the outcomes achieved by experiment however, both of them show a proximate trade line. Sincere observation over the plot can manifest that the values obtained by the current study are in between the experimental and numerical values of the mentioned paper. Noticeable steep increment is there for the numerical analysis of the present paper at elevated applied voltage whereas it maintains a relatively similar slope for lower magnitude of applied voltage. Nevertheless, the graph showcases meditating values of total volumetric flow rate for the same range of applied voltage due in other paper and so it provides necessary acceptance for the computational system to trust on and move forward. All the necessary numerical conditions applied for the validation of the work is tabulated in Table 3.

**Table 3:** Applied numerical conditions for validation purpose^40^.

Working fluid	PBS (Phosphate buffered saline)
Magnetic flux density *B*_*z*_	0.32T–0.5T
Initial temperature *T*_*o*_	300 K
Applied electric current *I*	0 mA–30 mA
Time step $\partial t$	$5 \times {10}^{-6}$ s

### Declaration of generative AI and AI-assisted technologies in the writing process

During the preparation of this work the authors used Quillbot (Paraphrasing tool) in order to make the writing more coherent and up to the standard of scientific community. After using this tool, the authors reviewed and edited the content as needed and take full responsibility for the content of the publication.

## Result and discussion

In this section, we describe the impact of three distinct blood-based hybrid nanofluids (namely Pt, Au, and MWCNT) on the fluid, magnetic, and electrical properties of the MHD micropump, specifically in relation to radiation. The study aims to investigate the numerical outcomes of velocity distribution, pressure drop, and temperature gradient along the flow path of the micropump for various nanofluids. The investigation is conducted based on two dimensionless parameters, namely Rd and Ha. The parameter known as radiation number (*Ra*) is used to quantify the thermal radiation emitted by radioactive fluids utilized as a transport medium in scientific studies. Similarly, the Hartmann number (*Ha*) is a measure of the ratio of electromagnetic force to the viscous force of hybrid bio-convective nanofluids. Based on two parameters, the MHD micropump's fundamental properties, namely MFD (*magnetic flux density*) and EFI (*electric flux intensity*), are analysed alongside fluid properties. The data is presented in three different visualization techniques. Line and surface plots are utilized in scientific visualization to represent the outcomes of data analysis. They are particularly useful in displaying the distribution of data points along the surface of a pump. In this study, volume plots are availed to gain insight into the behaviour of nanofluids as media for transport in the microchannel of a micro pump. Finally, based on the performance of the chosen bioconvective nanofluids, an optimization operation is performed, and the best candidate is chosen for additional research in radiative oncological support with a number of conclusions.

### Effect of radiation on average velocity distribution along flow channel of the MHD micropump

**Figure 7:**
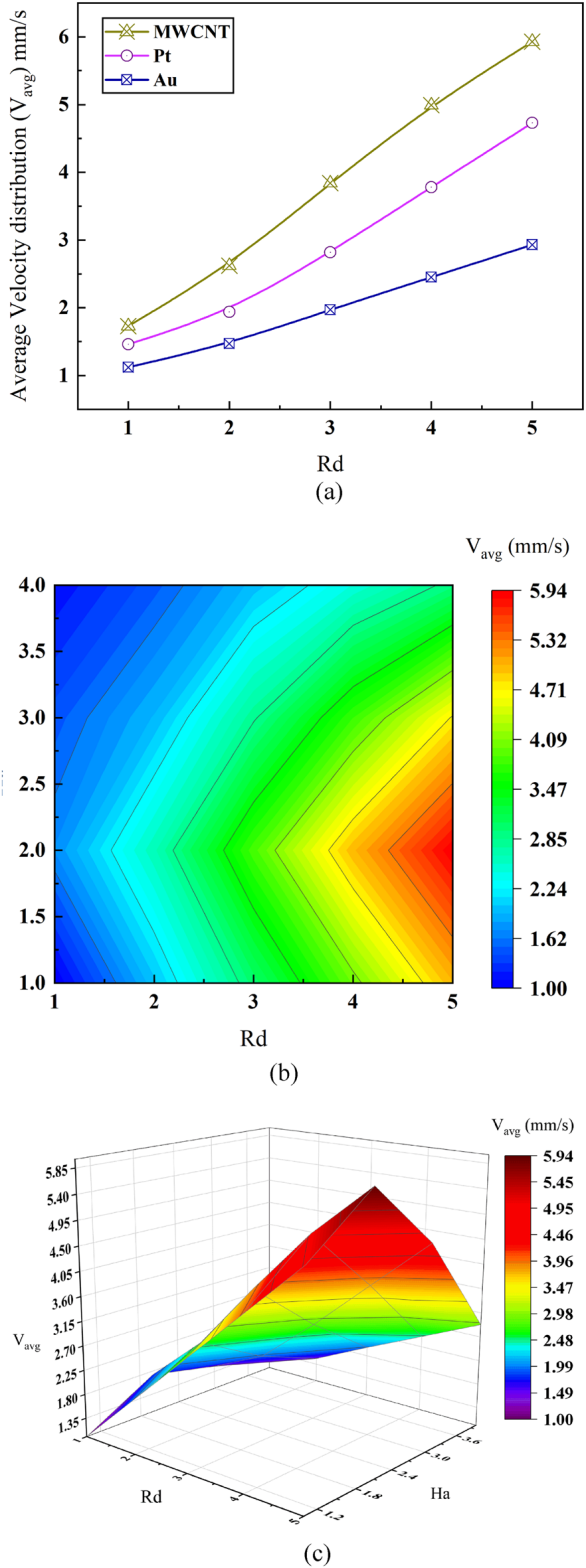
Effect of Radiation number *Rd* and Hartmann number *Ha* on average velocity distribution.

Impact of radiation number (*Rd*) and Hartmann number (*Ha*) on average velocity distribution through micro channel of the MHD micropump for blood based—Pt, Au and MWCNT nanofluids has been discussed in this subsection. Figure 7a provides line plot to understand the relative performance of the mentioned plasma based nanofluids for a range of Rd values. Figure shows that all the nanofluids achieve maximum average velocity for maximum radiation number of 5 presenting MWCNT, Pt and Au with magnitude of 6 mm/s, 5.85 mm/s and 2.92 mm/s respectively. Among the presented plasma cored nanofluids MWCNT exhibits steep rise with respect to increasing Rd values while Au shows more linear increment for the same. However, Pt showcases a moderate accretion of velocity with Rd values ranging from 1 to 5. Considering the three nanoparticles, MWCNT seems to be influenced by Rd values mostly as it shows a protean increment of quadratic nature while Au is to be least as it maintains linearity all through different values of Rd. As rapid transportation of plasma through human veins and arteries will result in rapid action of the intravenous therapy (IV therapy) as well as rapid radiative action during dealings with cancer cells, the most responsive plasma cored nanoparticles are desired. And so MWCNT proves to be the one with swift oncological feedback with placing Pt as next to it. Although Au shows positive attribute with increase of Rd number, it still lacks the response speed as well as the velocity magnitude like the other twos and therefore might be the least choice in this case.

Figure 7b signifies the variation of Hartmann number (Ha) for different regimes of average velocity distribution along with changing Rd values for selected blood based MWCNT nanofluid. Sincere look over the contour plot can reveal the fact that the average velocity distribution is much lower for low radiation value even though the viscous and electromagnetic force are higher. For example—velocity through the microchannel maintains a lower magnitude of around 1.00 mm/s to 2.24 mm/s for Rd ranging from Rd = 1 to Rd = 3 even though Ha = 4. The contour plot illustrates that average velocity of the microchannel along different values of Ha number does not change much except at relatively lower value of Ha. Nanofluids with extreme velocity can be found for maximal value of Rd which is 5 and also at relatively lower value of Ha varying from Ha = 1 to Ha = 2.5 while it becomes moderate when Ha value goes from 2.6 to 4. Such excerpt can be deduced that at overall view—velocity distribution along the microchannel of MHD pump is largely dominated by Rd number whereas lower Ha value can impact the velocity profile minorly as such can be seen in the contour diagram. Figure 7c details a more comprehensive view of the interrelationship among Ha (electromagnetic force to viscous force), Rd (radiative behaviour) and V_avg_ of the used nanofluids in three-dimensional plot. From this volume plot it can be seen that maximum average velocity of 5.94 mm/s is observed at Rd = 5, Ha value ranging in between Ha = 1.8 to Ha = 2.4. Understanding the line, surface and volume plots—it can be said that for precipitated result in radiative oncology, that is to say for faster transportation of blood fused medications, the velocity can be properly controlled at higher radiative effect while maintaining a lean electromagnetic effect.

### Effect of radiation on average pressure drop along flow channel of the MHD micropump

**Figure 8:**
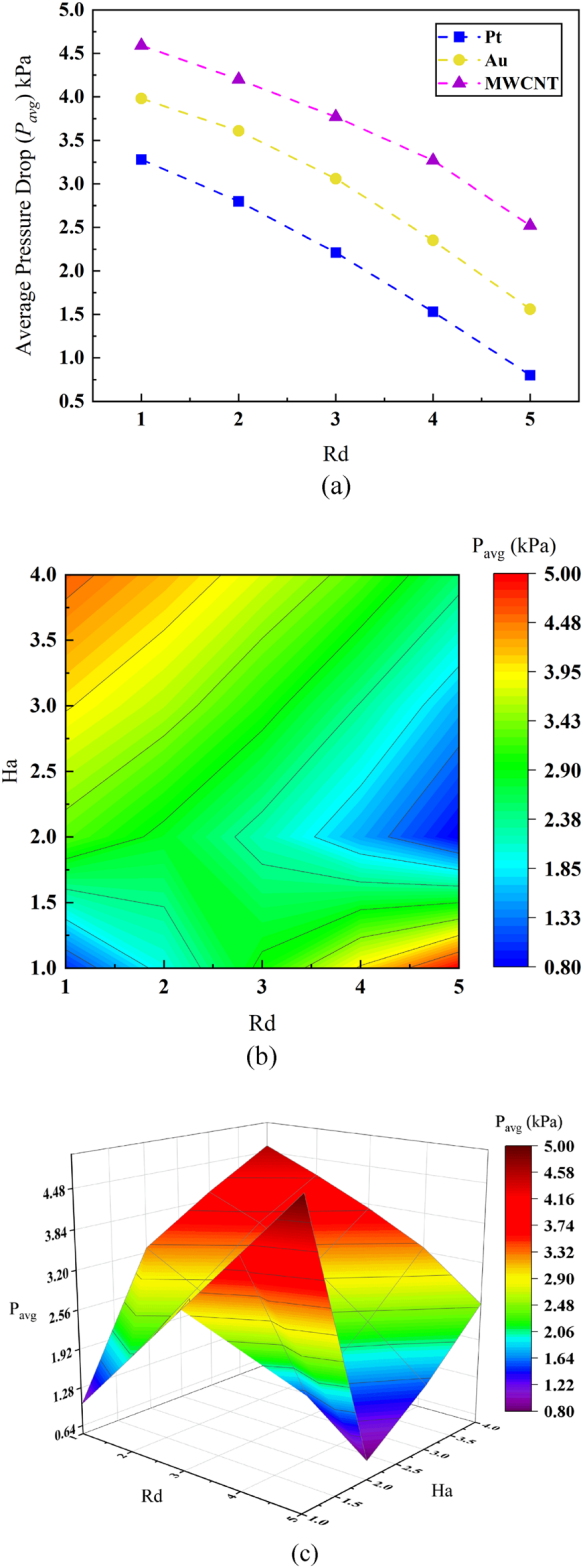
Effect of Radiation number *Rd* and Hartmann number *Ha* on average pressure drop.

A line plot including relative performance of three different blood based nanofluids is demonstrated in Fig. 8a Numerical outcomes reveal that Pt, Au and MWCNT suffers notable pressure drop of 77% (3.25–0.5 kPa), 56.35% (4.00–1.75 kPa) and 34% (4.5–2.75 kPa) as per their relative merits. Increasing pressure loss will demand increasing pumping power for the pump and thereby elevated Lorentz force to operate. However, for MHD pump of micro size it is not much cost worthy as well as pretty intricating to generate sufficient electromagnetic force to generate the demanding Lorentz force. Due to this reason MWCNT associated blood based nanofluid is supposed to be the most desired transporting medium to incorporate in MHD pump among all the three nanofluids even though all of them need necessary inflated input pressure to flow through the microchannel. At the same time Pt nanoparticles would be a choice for its relatively less initial pressure requirement compared to the other ones however does not necessitate due to its associated larger pressure drop in the pump channel.

To provide particular focus on the pressure drop of blood based MWCNT nanofluid—a two-dimensional (surface plot) and three-dimensional (volume plot) contour plots simplifying inter correlation of two dimensionless parameters—Ha and Rd upon average pressure drop P_avg_ are provided in Fig. 8b and c respectively. From the surface plot it can be excerpted that maximum pressure drop along the microchannel occurs for maximum Rd value with least Ha value (Rd = 5 and Ha = 1) and maximum Ha value with minimum Rd value (Ha = 4 and Rd = 1). However, none of these maximal value sustains as the minor parameter starts to increase *i.e.,* pressure drop initiates to drop as Ha ranges from Ha = 0.5 to Ha = 3.25 for Rd = 5. This could be due to stronger electromagnetic force (externally applied) and weaker viscous force (internal force of nanofluids) that dampens strong radiative flux within the nanofluid particles and thereby provides just enough Lorentz force. Similarly, pressure loss reduces as Rd proteins from Rd = 2 to Rd = 5 for Ha = 4. One interpretation could be excessive electromagnetic force dominance the flow at lower Rd value as it creates more friction at the solid liquid interface of the microchannel for which it ultimately ceases to a larger pressure drop. Accounting both of these phenomena a good understanding could be—pressure drop in the microchannel of the MHD pump will be dominated neither by extreme radiative nature of the selected nanofluids nor by their necessary electromagnetic force rather by a moderate combination of both parameters.

### Effect of radiation on average temperature gradient along flow channel of the MHD micropump

**Figure 9:**
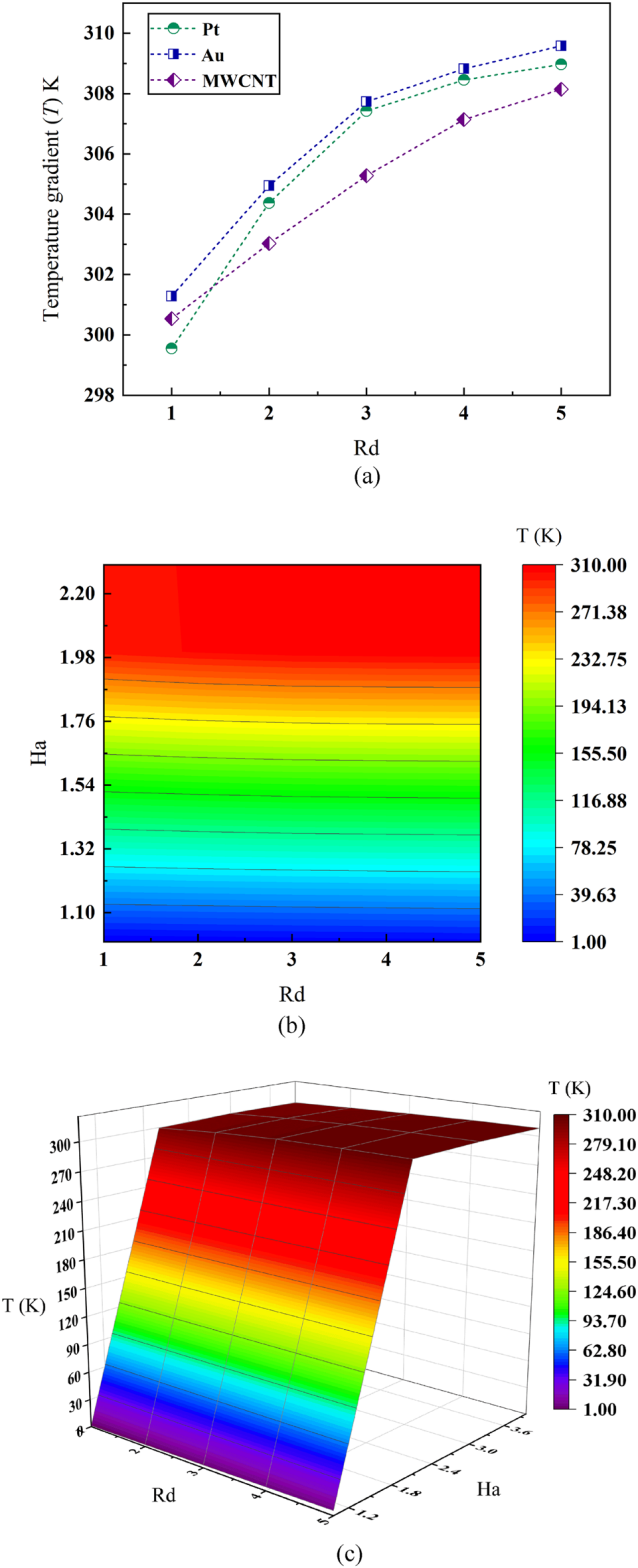
Effect of Radiation number *Rd* and Hartmann number *Ha* on average temperature drop.

The line plot of Fig. 9a showcases comparative temperature gradient performance analysis for three different plasma based nanofluids. Maximum temperature 309 K is observed for Au nanoparticles while Pt and MWCNT follows it with values of 308 K and 307 K. However, the scenario is different when it comes to temperature gradient along the channel wall as it is found that Pt nanoparticles provide maximum temperature gradient with relative value of 2.01% while Au seconds it with a relative value of 1.95%. The least value is obtained for MWCNT nanoparticles associated blood flow which is 1.38% on a relative merit. Electrical and electronic equipments suffer dysfunctionality if heat is not properly dissipated along the surface and so MHD micropump on the occasion of non-homogeneous heat distribution will be inhibited to work properly and ceased to fail. Higher temperature gradient follows less consistent heat dispersion and so making the pump more vulnerable to sudden burn out and poor performance. Considering overall performance of the three nanofluids—MWCNT is might be the one to put focus on as it provides the least thermal non-uniformity and thereby smoother cooling of the pump which ultimately corresponds to zero demand of complex cooling mechanism.

Further characteristics of average temperature gradient of such bioconvective MWCNT nanofluid have been emphasised in Fig. 9b and c as they present two-dimensional and three-dimensional contour plots for understanding the variation of externally applied electromagnetic force with respect to their radiative nature and average temperature gradient along the flow path. The figures suggest that temperature gradient for the MHD micropump is a strong function of Hartmann number Ha as it is seen that—protean value of Ha number from Ha = 1.76 to Ha = 2.20 corresponds to maximum average temperature change while it is being moderate for Ha = 1.45 to Ha = 1.76. A good observation over the contour plot (surface) will refer that temperature gradient has little to no effect due to the change in Rd value. The magnitude of temperature difference remains same irrespective of Rd column whereas it only changes with respect to dominant change in Ha number. One of the reasons behind this phenomenon could be the strong electromagnetic force applied externally that couples with the nanofluids’ internal magnetic properties to generate higher temperature variance. Due to such it is to be advised that—a lean to moderate electromagnetic force is to be incorporated to operate the MHD micropump as otherwise it might result in non-uniform heat transfer and thereby causing a fluctuating temperature gradient through the micro sized channel. The volume contour plot in Fig. 9c evinces the same behaviour of the temperature gradient as can be seen in the surface plot however provides a more in-depth analysis. The three-dimensional contour plot refers that for Ha = 1.8 to Ha = 2.6 average temperature gradient is maximum ranging from 210 to 310 K while it is mostly indifferent to Rd values even though a linear regressive relationship is maintained among the all three parameters.

### Effect of radiation on average magnetic flux density along flow channel of the MHD micropump

**Figure 10:**
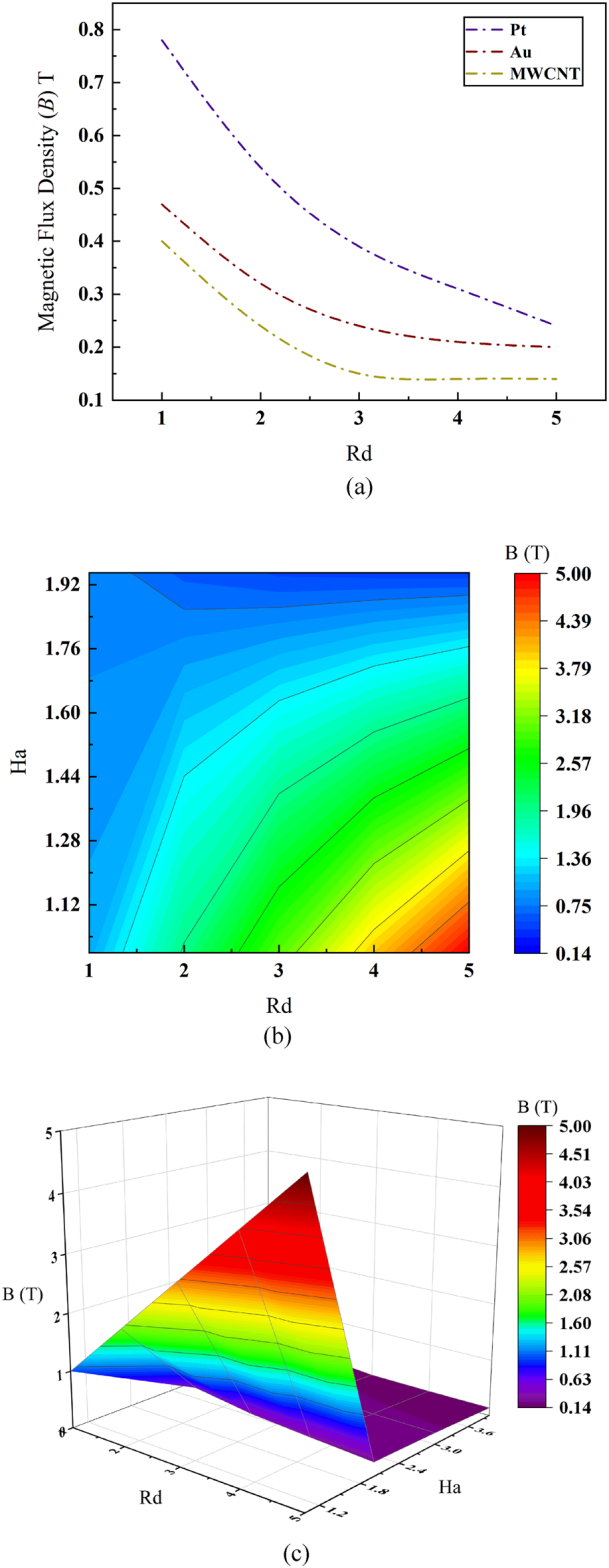
Effect of Radiation number *Rd* and Hartmann number *Ha* on average magnetic flux density.

Figure 10a illustrates the line plot of average magnetic flux density of different nanofluids. The relevant figure shares a negative intercorrelation of magnetic flux density with respect to Rd value which means radiative flux influences the magnetic flux negatively and so higher value of Rd will turn out for lower value of average magnetic flux density. Under this criterion, highest magnetic flux fluctuation occurs for Pt, a moderate for MWCNT and least for Au. Although the variance of flux magnitude is highest for Pt, the relative flux density for lower Rd values is also highest for Pt which intuits a smother electromagnetic force generation. The magnitude is relatively lesser for Au and MWCNT with a little difference in between them. Due to relatively moderate demand of strength of magnetic flux density with a more constant flux fluctuation, Au can be preferred as the desired nanofluid among the proposed one while MWCNT and Pt following it.

The surface contour plot in Fig. 10b shares a comprehensive mutual dependence among electromagnetic force to viscous force ratio (Ha), radiative flux (Rd) and magnetic flux density (B) for blood based Au nanofluid. It is to be seen from the plot that higher value of Rd (Rd = 4, Rd = 5) and comparatively lower value of Ha (Ha = 1, Ha = 1.28) result in maximum average magnetic flux density. On the other hand, for the same radiative value, the average magnetic flux density falls as the Ha value increases from Ha = 1.28 to Ha = 1.92. And furtherly, highest value of Ha number corresponds to a higher value in electromagnetic pulsation which should generate in a stronger magnetic flux intuitively. However, the phenomena is not such and one reason for this could be the sheer dominance of radiation number which is not allowing the increased electromagnetic force to influence magnetic flux density. Under this concept, it might not be wronged to assume that—the overall magnetic flux density over the surface of MHD microchannel is mostly governed by radiative nature of the plasma based nanofluids. This hypothesis is further integrated by volume plot of the microchannel in Fig. 10c that signifies ranges of maximum average magnetic flux density with respect to Rd = 3 to Rd = 5 and Ha = 1.29 to Ha = 1.88. Magnetic flux density reaches peak value of B = 4 T within that range while remains stable for higher Ha values that even shows indifference of B_avg_ towards Ha number at higher Ha values while Rd value is also higher.

### Effect of radiation on average electric flux intensity (EFI) along flow channel of the MHD micropump

**Figure 11:**
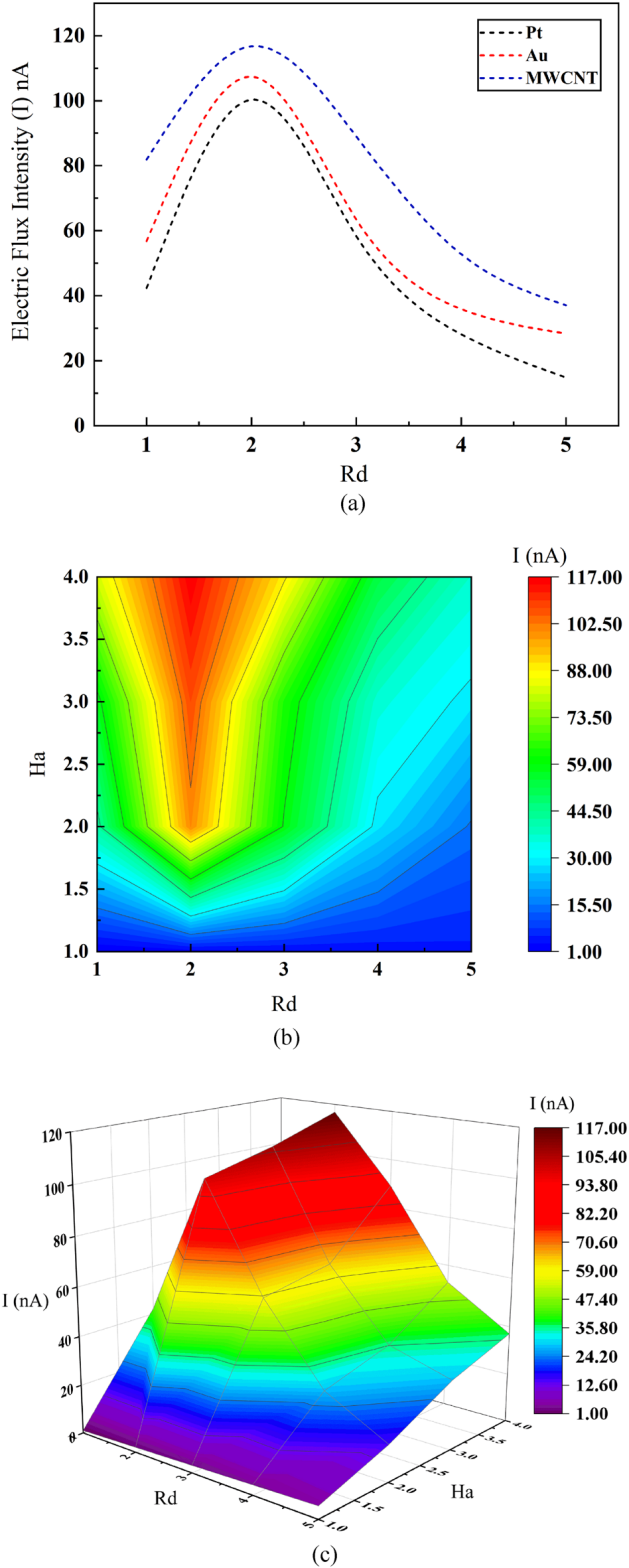
Effect of Radiation number *Rd* and Hartmann number *Ha* on average electrical flux intensity.

Average electrical field intensity (I_avg_) indictes the electrical field behvior of the nanofluids within microchannel. Figure 11a illustrates such behavior with respect to increasing Rd values for three different blooad based nanfluids. The electrical field intensity vs Rd graph resembles “bell curve” or “normal distribution curve” where each nanofluids reach a maximum value and then starts to drop to a certain point. For the electrical flux intensity MWCNT has maximum electrical flux with a value of 119 nA and Au and Pt proceeds it with maximum values of 107 nA and 91 nA respectively. However, for proper operation of the micropump minimal electrical flux droppage is also important and from the figure it is to be noted that maximal drop in electrical flux occurs for Pt whereas it is lesser for MWCNT and least for Au with values of 40%, 23% and 19% respectively. A better leverage for this prospect of smooth blood flow through sophisticated part of human body would be to choose a nanofluid with optimal electric flux intensity while a moderate to less fluctuation in electrical flux intensity. Considering this—Au is thought to be the desired one among the three due to its moderate electrical flux intensity with least droppage in it.

Figure 11b provides the contour plot of the MHD microchannel with respect to electromagnetic force ratio with viscous force (Ha), radiation prameter (Rd) and average electrical flux intensity (I_avg_) for the eearlier specified desired plasma based Au nanofluid. It can be signified from the plot that—maximum value of electrical flux intensity is observed for Rd = 2 and for the range of Ha = 2.00 to Ha = 4.00 which means that the electral flux is more observable when the radiative nature of the nanofluid is less expressive whereas electromagnetic force is more dominant. As stronger electromagnetic force is proportinal to stronger electrical field strength and intensity—one of the sole “could be” reasons behind this phenomena. However, at higher Rd value minimum electrical flux intensity is seen and there is minimal to no change along the variance of Ha value. Reversal of electromagnetic force due to greater radiative sorrounding might be the cause for poor electrical flux generation. Understanding this scenario concluson can be added that at proteaned radiation flux (higher value of Rd), electrical flux is mostly dominanted by Rd parameter and flux generation is least. However, a moderate to lower value of Rd (Rd = 1 to Rd = 2) along with higher range of Ha value is sufficient to generate sufficient magnitude of electrical flux as lower radiation flux prompts average electrical flux intensity to be dependent on Ha value and thereby higher electromagnetic force. An in depth and more focused study of the above mentioned is illustrated in Fig. 11c that shapes up the relationship among the three parameters—a span of high electrical flux intensity (101 nA ~ 117 nA) is observed for Rd = 2 to Rd = 3 while Ha value ranges from Ha = 2 to Ha = 4.

## Conclusion

The research concentrates on the radiative behaviour of blood-based biological nanofluids—Pt, Au, and MWCNT—when used in a MHD micropump. In terms of fluidic properties—temperature gradient, velocity distribution, pressure drop, magnetic property—magnetic flux density, and electrical property—electrical flux intensity, a qualitative analysis of the proposed topic has been conducted. The ultimate results of the study can be summarized as follows:

- MWCNT and Pt are found to be the most efficient transporting bioconvective nanoparticles due to their smooth responsive nature at high radiative condition. Average velocity of the nanofluids through microchannel is mostly governed by radiation nature of its kind and a combination of high radiation with moderate electromagnetic force is prompted for better plasma transportation.
- Average pressure drop curve demonstrates that MWCNT based blood flow is the most desired nanoparticle among the selected three due to its sufficient intake pressure as well as minimal pressure drop along the channel. The other surface and volume plots reveal that—maintaining coherence in between the radiation flux and electromagnetic flux is the key for desired (minimal) pressure drop as doing such will reduce the required pumping power.
- Comparison of propagated thermal gradient among the nanofluids provides—MWCNT is the nanofluid with least temperature variance and thereby a uniformed dissipation of heat is more likely to be achieved for this nanoparticle fused with blood. Interrelationship surface and volume plot focuses that—a leaner or moderate electromagnetic force is advised for better thermal regulation along the pump channel and thereby a more smoother drug delivery during different heat sensitive intravenous medication.
- Effective magnetic flux density grid test showcases that—Au nanoparticles when conducts in radiative environment provides maximum magnitude of magnetic flux; even when the radiation value is very high. Surface and volume plots showpiece that—magnetic flux for the nanofluids maintains a highly proportional relationship with the emitted radiation. This phenomenon is to be noted to leverage using effective radiative nanofluids for IV embedded chemotherapy or any type of cell treatment.
- Average electrical flux intensity analysis discloses that Au provides a moderate flux intensity while maintaining very minimal flux droppage contrary to the other nanofluids. However, at higher radiative environment the electrical flux is prompted by Ha value which is the ultimate electromagnetic force. This understanding of the electrical behaviour of the nanofluids can be transformed to precipitate a more effective oncological cure of cancerous disease.

Considering the abovementioned outcomes, it can be stated that – MWCNT along with Au came out as the two superior nanofluids to be incorporated in advanced radioactive oncological treatment for future purpose. In addition to conclusive statements, further exploration can be possible in the following scopes –

- The study is based on numerical formulation and solution of the computational domain. Therefore, experimental analysis is most welcomed to develop the issue any better.
- FEA analysis of the project is done focusing radiation characteristics of the MHD micropump model and so two dimensionless parameters are taken into consideration. Mixed convection within the micropump can be further investigated by parameters such as—Sherwood number (*Sh*), Richardson number (*Ri*) etc. against the Radiation number (*Rd*).
- Power-law model of MHD using nanofluids is negotiated in the paper due to related complexity in computational process and time, however it remains a good prospect to advance in future (Supplementary Appendix).

### Supplementary Information

Supplementary Information.

## Data Availability

The datasets generated and/or analysed during the current study are not publicly available due to confidentiality of the research raw data but are available from the corresponding author on reasonable request.
