# Supplementary material for: Enhancing radiative efficiency in MHD micropumps using plasma-infused hybrid bioconvective nanofluids for advanced radiative oncology at tertiary level
Source: Sci Rep. 2023 Oct 27;13:18452. doi: 10.1038/s41598-023-45513-5 (PMC10611780; doi:10.1038/s41598-023-45513-5)
Supplement: Supplementary file 1 — Supplementary Information. [file 41598_2023_45513_MOESM1_ESM.docx]

**Appendices**

| $MHD$ | Magneto Hydrodynamic |
| --- | --- |
| $BL$ | Blood |
| $Au$ | Gold nanoparticles |
| $Pt$ | Platinum nanoparticels |
| $MWCNT$ | Multi walled carbon nanotube nanoparticles |
| $MFD$ | Magnetic field density |
| $EFI$ | Electric field intensity. |
| $Rd$ | Radiation parameter |
| $Ha$ | Hartmann number |
| $Al$ | Aluminum |
| $x,y,z$ | Dimensional cartesian coordinates |
| $\rho$ | Density |
| $D$ | Electric displacement field |
| $B$ | Magnetic field |
| $E$ | Electric field |
| $H$ | Magnetic field intensity |
| $J$ | Electric current density |
| $t$ | Time |
| $\nabla$ | Vector differential operator |
| $u,v$ | Velocity components |
| $nf$ | Nanofluid |
| $\mu$ | Dynamic viscosity |
| $P$ | Pressure |
| $T$ | Temperature |
| $\alpha$ | Thermal diffuisivity |
| $\vartheta$ | Kinematic viscosity |
| $q$ | External heat flux |
| $C_{p}$ | Reynolds number |
| $C$ | Cold system |
| $K$ | Thermal conductivity |
| $\varphi$ | Non dimensional temperature |
| $V$ | Applied electrical voltage |
| $L$ | Length of pump |
| $U, V$ | Dimensionaless velocity components |
| $N$ | Any direction |
| $X,Y$ | Dimensionless cartesian coordinates |
| $avg$ | Average value |
| $\beta$ | Thermal expansion coefficent |
| $s$ | Solid |
| $f$ | Fluid |
| $Pr$ | Prandtl number |
| $I$ | Electric current |
| $\eta$ | Dimensional optical thickness |
| $\sigma$ | Electrical conductivity |
| $in$ | Inlet |
| $out$ | Outlet |
